# Assessing safety and treatment efficacy of running on intervertebral discs (ASTEROID) in adults with chronic low back pain: protocol for a randomised controlled trial

**DOI:** 10.1136/bmjsem-2022-001524

**Published:** 2023-01-17

**Authors:** Scott D Tagliaferri, Daniel L Belavy, Steven J Bowe, Matthew J Clarkson, David Connell, Emma A Craige, Romina Gollan, Luana C Main, Clint T Miller, Ulrike H Mitchell, Niamh L Mundell, Christopher Neason, Claire L Samanna, David Scott, Jamie L Tait, Grace E Vincent, Patrick J Owen

**Affiliations:** 1 Deakin University, Institute for Physical Activity and Nutrition (IPAN), School of Exercise and Nutrition Sciences, Geelong, Victoria, Australia; 2 Hochschule für Gesundheit (University of Applied Sciences), Department of Applied Health Sciences, Division of Physiotherapy, Gesundheitscampus 6-8, 44801, Bochum, Germany; 3 Deakin University, Biostatistics Unit, Faculty of Health, Geelong, Victoria, Australia; 4 Victoria University of Wellington, Wellington, New Zealand; 5 Institute for Health and Sport, Victoria University, Melbourne, Victoria, Australia; 6 Imaging @ Olympic Park, AAMI Park, 60 Olympic Boulevard, Melbourne, Victoria, Australia; 7 Medical and Applied Sciences, Central Queensland University, Rockhampton, Queensland, Australia; 8 Medical Psychology, Neuropsychology and Gender Studies & Center for Neuropsychological Diagnostics and Intervention (CeNDI), Faculty of Medicine and University Hospital Cologne, University of Cologne, Cologne, Germany; 9 Department of Exercise Sciences, Brigham Young University, Provo, Utah, USA; 10 School of Clinical Sciences at Monash Health, Monash University, Clayton, Victoria, Australia; 11 Appleton Institute, School of Health, Medical and Applied Sciences, Central Queensland University, Adelaide, South Australia, Australia

**Keywords:** physical therapy, physiotherapy, MRI, physical activity, spine

## Abstract

Poor intervertebral disc (IVD) health is associated with low back pain (LBP). This 12-week parallel randomised controlled trial will evaluate the efficacy of a progressive interval running programme on IVD health and other clinical outcomes in adults with chronic LBP. Participants will be randomised to either a digitally delivered progressive interval running programme or waitlist control. Participants randomised to the running programme will receive three individually tailored 30 min community-based sessions per week over 12 weeks. The waitlist control will undergo no formal intervention. All participants will be assessed at baseline, 6 and 12 weeks. Primary outcomes are IVD health (lumbar IVD T2 via MRI), average LBP intensity over the prior week (100-point visual analogue scale) and disability (Oswestry Disability Index). Secondary outcomes include a range of clinical measures. All outcomes will be analysed using linear mixed models. This study has received ethical approval from the Deakin University Human Research Ethics Committee (ID: 2022-162). All participants will provide informed written consent before participation. Regardless of the results, the findings of this study will be disseminated, and anonymised data will be shared via an online repository. This will be the first study to evaluate whether a progressive interval running programme can improve IVD health in adults with chronic LBP. Identifying conservative options to improve IVD health in this susceptible population group has the potential to markedly reduce the burden of disease. This study was registered via the Australian New Zealand Clinical Trials Registry on 29 September 2022 (ACTRN12622001276741).

**WHAT IS ALREADY KNOWN ON THIS TOPIC**

- Exercise training is known to improve a range of health-related outcomes (eg, pain intensity and disability) in adults with chronic low back pain.

**WHAT THIS STUDY ADDS**

- This randomised controlled trial will be the first to examine the safety and efficacy of a progressive interval running programme on intervertebral disc (IVD) health.

**HOW THIS STUDY MIGHT AFFECT RESEARCH, PRACTICE OR POLICY**

- This study represents a critical step towards identifying a conservative treatment capable of improving IVD health in this susceptible population group.

## Introduction

Low back pain (LBP) is the leading cause of age-standardised disability worldwide and affects approximately 7.1% (568.4 million) of the global population.^1^ Annual healthcare expenditure associated with LBP is more than US$134.5 billion in the USA^2^ and $A9 billion in Australia.^3^ Given that as much as 85% of LBP healthcare expenditure stems from chronic LBP (CLBP; ≥12 week pain duration),^4^ identifying effective management strategies is vital.

We previously showed via a network meta-analysis^5^ that exercise training effectively reduces pain intensity and disability in adults with CLBP. However, only some types of exercise training (eg, Pilates, motor control exercises and resistance training) resulted in clinically meaningful improvements in both pain intensity and disability.^6^ These types of exercise training primarily target paraspinal musculature, which suggests improving spinal tissue health can reduce pain intensity and disability.^6^ However, limited studies to date have examined the effect of exercise training on other spinal tissues, such as the intervertebral disc (IVD).

A meta-analysis of 14 studies (3097 participants)^7^ showed individuals with MRI-derived markers of poor IVD health (eg, IVD bulge, degeneration, extrusion or protrusion) were up to eightfold more likely to have LBP. Poor IVD health may lead to surgery associated with additional risks and high financial costs.^8 9^ Identifying conservative interventions that improve IVD health in adults with CLBP is critical for reducing individual and healthcare burdens.

We previously conducted the first exercise training randomised controlled trial designed to assess the possibility of improving IVD health in adults with CLBP.^10^ The intervention group received 6 months of combined resistance and cardiovascular exercise training (two 60 min supervised sessions per week) designed to mechanically stress IVDs. The control group also received treatment (motor control exercises and manual therapy), designed to minimise exercise-related mechanical stress to IVDs. While no between-group differences in IVD health were observed, exercise training led to within-group changes in two MRI-derived markers of IVD health: (1) IVD T2 and (2) IVD height expansion in short-duration lying. Both of these observations indicated that exercise training might be viable for improving IVD health,^11^ yet the lack of between-group difference suggests different types of exercise training require investigation.

Building on our prior data,^12^ we identified that a history of cardiovascular exercise training,^13^ and specifically cardiovascular exercises performed in an upright posture (eg, running, rather than swimming or cycling),^14^ were associated with better IVD health. Our observations support that improving IVD health appears contingent on the type of exercise training provided. Similar to how impact exercise training (eg, jumping) is required to improve bone health,^15^ cardiovascular exercise training in an upright posture (eg, running) appears integral to improving IVD health.^11^ Therefore, the primary aim of this 12-week single-blind (researchers) two-arm parallel individually randomised controlled trial is to determine the efficacy of a progressive interval running programme on IVD health, pain intensity and disability in adults with CLBP. The secondary aims are to determine intervention feasibility and efficacy across a range of subjective and objective health-related outcome measures.

## Methods

### Study setting

Data will be collected in a dedicated room at an imaging facility (Imaging @ Olympic Park, Melbourne, Australia), at a commercial pathology clinic collection centre (Melbourne Pathology, Melbourne, Australia) and via an online questionnaire (REDCap, Nashville, USA).^16 17^ The intervention will be conducted within the community of the participants.

### Patient and participant involvement

Patient and participant involvement will be planned, recorded and evaluated throughout the trial according to the Guidance for Reporting Involvement of Patients and Public shortform checklist.^18^

### Eligibility criteria

We will recruit a sample of 40 adults (18–45 years) with non-specific (no known specific pathology) chronic (>12 weeks) LBP (pain located between the costal margin and above the inferior gluteal folds, with or without leg pain and experienced on most days in an average week). Exclusion criteria will consist of: (a) history of spinal surgery, spine trauma (eg, fracture or car accident), cauda equina symptoms, known structural scoliosis requiring surgical consultation, symptomatic radiculopathy (diagnosed via medical professional or leg pain greater than back pain), inflammatory spondyloarthropathies or non-musculoskeletal causes of LBP (eg, infection), (b) inability to communicate in English, (c) pregnancy, lactating or less than 1-year postnatal, (d) current or prior elite athletes (eg, member of Australian Institute of Sport, State Institutes or Academies of Sport or the national squad of any sport),^19^ (e) any absolute contraindications for MRI, (f) any absolute contraindications for exercise training, (g) participation in running or sport that involves running in the last 3 months (>1 session per month), (h) having experienced a lower limb injury in the last 6 weeks, (i) deemed higher risk of adverse event due to physical activity per the Adult Pre-Exercise Screening System^20^ and (j) unable to access a smartphone with a cellular internet connection.

### Interventions

#### Exercise training

The exercise training will consist of three community-based 30 min sessions per week for 12 weeks prescribed by an accredited exercise physiologist from the research team. Exercise training sessions will be individualised based on information during a 30 min initial assessment completed at Imaging @ Olympic Park. Exercise training sessions will consist of a 5 min general warm up followed by an interval-based running programme that will be progressed weekly as guided by an accredited exercise physiologist from the research team. The programme for each participant will be delivered and monitored through the RunKeeper application (ASICS Runner App, Boston, USA), using a preallocated study code allowing for deidentified tracking of running speed, distance and surface (ie, grass, gravel, paved, trail or mixed). Participants will receive educational content delivered via REDCap throughout the intervention covering the following topics: (a) ideal running speed, (b) footwear selection, (c) the safety of running and (d) dealing with setbacks (online supplemental appendix A). Participants will initially meet for 15 min weekly (weeks 1–4) and then fortnightly (weeks 6–12) via secure video consultation (Zoom Video Communications, California, USA) with a member of the study team to discuss the exercise training programme.

10.1136/bmjsem-2022-001524.supp1 Supplementary data

The exercise programme will consist of short running intervals interspersed with rest periods of walking (table 1). This interval-based training is an effective way to increase running capacity in untrained novice runners^21^ and is recommended following hip and lower limb injuries to safely return to running.^22–24^ Participants enter the interval training programme at stage 1, 2 or 3 as determined by a 2 min running tolerance test at the initial physical assessment. During this run test, participants will be instructed to run at a slow to moderate pace for as long as they are comfortable, up to a maximum of 2 min. Participants who can complete the slow run comfortably for: (a) 0–44 s will start at stage 1 of the programme; (b) 45–89 s will start at stage 2 of the programme; and (c) 90–120 s will start at stage 3 of the programme. Participants may choose their starting point (stage 1, 2 or 3) in consultation with the accredited exercise physiologist, irrespective of the test results. At each stage, participants can self-select their chosen number of repeats to complete based on their overall perceptions of exertion, breathlessness and pain intensity. For example, at stage 2, participants can select to complete between 6 and 10 repeats of 30 s running. This approach accounts for the daily fluctuations in pain intensity observed in up to 35% of individuals with CLBP^25^ while promoting self-efficacy through autonomy and shared decision-making.^26^ Once participants complete the upper repeat range each week, they progress to the next stage. To reduce the risk of injury due to the rate of progression, participants can only increase one stage per week and must complete at least two sessions per week before progressing to the following stage.^22^ The increase in average running time per week (table 1) is greater over the first 6 weeks and reduces throughout the programme. This is due to a conservatively chosen starting point to facilitate confidence with running, facilitate familiarity with sensations associated with muscle fatigue and reduce the interference of possible fatigue-related pain interpreted as injury pain. For example, the graded exposure to running commences at 1.5 min total running time per session at stage 1, progressing by 20%–100% over the first 6 weeks before levelling off to increases of approximately 10%–15% after that. Importantly, in novice runners, there is no difference in the prevalence of running-related injuries between training programmes that adhere to a strict 10% weekly increase and those that include greater weekly progressions of up to 50%.^27^ If participants reach the final stage before the end of the intervention, they will stay in this stage for the remaining weeks. Throughout the intervention, participants will be advised to run at a slow to moderate pace (maximum of 10 km per hour or 6 min per km), which corresponds with velocities thought to have a positive stimulus on the IVD.^12^ In collaboration with the accredited exercise physiologist, participants can regress to an earlier stage of the interval programme if deemed necessary (eg, significantly increased LBP, other injuries/soreness, following periods of poor adherence).

**Table 1:** Interval training programme

Stage	Run interval (s)	Walk interval (s)	Repeats		Total session time (min)		Total running time per session (min)		Total running time per week (min)	Increase in weekly running time from the previous week
			Lower	Upper	Minimum	Maximum	Minimum	Maximum		
1	15	120	6	10	13.5	22.5	1.5	2.5	4.5	NA
2	30	120	6	10	15	25	3	5	9	100.00%
3	45	115	6	10	16	26.7	4.5	7.5	13.5	50.00%
4	60	90	6	10	15	25	6	10	18	33.33%
5	75	75	6	10	15	25	7.5	12.5	22.5	25.00%
6	90	60	6	10	15	25	9	15	27	20.00%
7	105	45	6	10	15	25	10.5	17.5	31.5	16.67%
8	120	45	6	10	16.5	27.5	12	20	36	14.29%
9	135	45	6	8	18	24	13.5	18	40.5	12.50%
10	150	30	6	8	18	24	15	20	45	11.11%
11	165	30	6	8	19.5	26	16.5	22	49.5	10.00%
12	180	30	6	8	21	28	18	24	54	9.09%
13	210	15	6	8	22.5	30	21	28	63	16.67%

Determined by baseline testing, participants start at stage 1, 2 or 3 and progress to the next stage once they complete the upper repeat range. The total running time per week is calculated based on 3 weekly sessions at the lower repeat range.

NA, not applicable.

#### Waitlist control

Participants allocated to the waitlist control arm will be asked to manage their LBP per usual (eg, general practitioner management, over-the-counter pharmacotherapy) and avoid commencing a running programme. No formal intervention will be provided during the 12-week follow-up. Following completion of the study, waitlist participants will be offered the same exercise training programme and one-on-one consultation with an accredited exercise physiologist as per the intervention group.

### Outcomes and data collection methods

All participants will be assessed at baseline, 6 and 12 weeks. The primary outcomes of this study are lumbar IVD T2, LBP intensity and disability. Secondary outcomes will examine IVD height and volume, paraspinal muscle volume and size, paraspinal and vertebral body fat fraction, feasibility, sleep quality, insomnia severity, health-related quality of life, inflammatory blood markers, habitual physical activity, recovery and treatment expectations, activity-specific beliefs, kinesiophobia, depression, anxiety and stress, pain catastrophising, pain self-efficacy and adverse events. Researchers processing and analysing MRI data will be blinded to group allocation. Given the participant is the outcome assessor for self-reported measures and the inability to blind group allocation, these outcomes will not be blinded. An overview of the data collection is available in table 2.

**Table 2:** Overview of the study processes and data collection

	Enrolment	Allocation/baseline	6-week follow-up	12-week follow-up
	−t_1_	t_0_	t_1_	t_2_
Enrolment				
Eligibility screen	X			
Informed consent	X			
Allocation		X		
Interventions				
Intervention		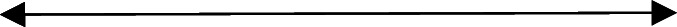		
Waitlist		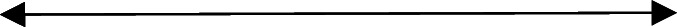		
Assessments				
Lumbar spine MRI		X	X	X
Pain intensity		X	X	X
Disability		X	X	X
Feasibility		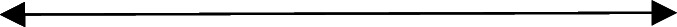		
Sleep quality		X	X	X
Insomnia severity		X	X	X
Health-related quality of life		X	X	X
Inflammatory blood markers		X		X
Habitual physical activity		X	X	X
Past exercise history		X		
Recovery and treatment expectations		X	X	X
Activity-specific beliefs		X	X	X
Kinesiophobia		X	X	X
Depression, anxiety and stress		X	X	X
Pain catastrophising		X	X	X
Pain self-efficacy		X	X	X
Social support		X	X	X
Pressure pain thresholds		X		X
Exercise-induced hypoalgesia		X		X
Adverse events		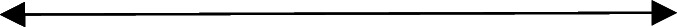		
Participant feedback				X
Physical assessments		X		

#### Lumbar spine MRI

The morphology of IVDs,^12^ vertebral marrow fat^28^ and paraspinal muscles^12^ will be assessed via MRI at baseline, 6 weeks and 12 weeks using the following sequences: (1) T2 Sag spin-echo multiecho, (2) Sag mDIXON, (3) Axial mDIXON and (4) T2-weighted Sag. Each sequence will be completed using established and reliable methods.^12 28^ These sequences will enable the quantification of IVD composition, vertebral fat fraction and paraspinal muscle size and composition. To avoid the impact of diurnal variation,^29^ all scanning will be performed after at least 4 hours since waking and participants will be instructed not to perform any strenuous physical activity or sport on the day of scanning. Immediately before scanning, participants will be required to sit for 20 min. Blinded image tracing (ImageJ: https://imagej.nih.gov/ij/) and analysis will be performed for all MRI outcomes as in prior studies by the team^12 28^ to acquire the following:

1. IVD T2 (primary outcome)^12^: Individual lumbar IVD T2 (in ms) will be calculated by a linear fit of the natural logarithm of the image intensity across the eight echo times of the T2 Sag spin-echo multiecho sequence. IVD T2 will be calculated for the entire IVD and five equidistant subregions (anterior annulus, anterior nucleus, centre nucleus, posterior nucleus, posterior annulus).^14^ Lumbar IVD T2 has excellent test–retest reliability (ICC=0.98).^30^
2. IVD height and volume^12^: Individual lumbar IVD height will be calculated from the T2 Sag spin-echo multiecho sequence. IVD volume will then be calculated by multiplying the area of each slice by the slice thickness and gap between slices.
3. Nucleus–annulus signal intensity ratio^14^: T2-weighted Sag sequences will be used to determine the signal intensity of five equidistant subregions (anterior annulus, anterior nucleus, centre nucleus, posterior nucleus, posterior annulus) to calculate the nucleus–annulus signal intensity ratio.
4. Paraspinal muscle volume and size^31^: Axial mDIXON sequences will be used to calculate muscle size and volume of the paraspinal muscles of multifidus, erector spinae, psoas major and quadratus lumborum. For these, we will primarily report on paraspinal cross-sectional muscle volume (cm^3^). Lumbar paraspinal muscle volume will be calculated by multiplying the area of each lumbar slice by the slice thickness plus the gap between slices. Lumbar level-specific average area (mm^2^) will also be calculated.
5. Paraspinal muscle fat fraction^31^: Axial mDIXON sequences will be used to calculate the paraspinal fat fraction. The fat fraction will be calculated as 100%×signal intensity fat/(signal intensity fat+signal intensity water).
6. Vertebral body fat fraction^28^: Sag mDIXON sequences will be used to calculate the vertebral body fat fraction. The fat fraction will be calculated as 100%×signal intensity fat/(signal intensity fat+signal intensity water).
7. IVD to vertebral body height ratio^14^: Sag mDIXON sequences will be used to calculate the vertebral body height. This will then allow for calculating IVD height to vertebral body height ratio.
8. Pfirrmann grading^32^: T2-weighted Sag sequences will be used to complete the radiographic grading of the IVDs using the Pfirrmann criteria.

#### Pain intensity

The visual analogue scale will quantify LBP intensity at baseline, 6 weeks and 12 weeks.^33^ The scale ranges from 0 to 100 points, with 0 considered no pain and 100 considered the worst pain imaginable. The primary measure will be the average pain intensity during the past week. We will also assess current pain intensity and worst pain over the last week as secondary measures. For adults with CLBP, a 20-point reduction will be considered the minimum clinically meaningful difference.^34^ The visual analogue scale has excellent test–retest reliability (ICC=0.90).^35^

#### Disability

The 10-item Oswestry Disability Index will quantify disability (poor physical function) due to LBP at baseline, 6 weeks and 12 weeks.^36^ Questions are rated from 0 to 5 points, with higher scores indicating greater disability. The total score from the questionnaire is doubled and represented on a 100-point scale. For adults with LBP, a 10-point reduction is considered the minimum clinically meaningful difference.^6^ The Oswestry Disability Index has good to excellent test–retest reliability (ICC=0.84–0.94).^36^

#### Feasibility

The following domains of feasibility will be documented throughout the study by members of the research team: recruitment ((1) enrolled participants compared with total screened potential participants, (2) reasons for ineligibility or declined participation, (3) enrolment timeline, (4) efficacy of recruitment pathways), attrition ((1) number of participants available for follow-up, (2) reasons for loss to follow-up), adherence ((1) overall training session attendance, (2) within-session training load completed), data collection ((1) missing data, (2) reasons for missing data), safety ((1) number of adverse events, (2) severity of adverse events), acceptability ((1) system usability survey). For feasibility purposes, additional pain intensity (visual analogue scale) measurements will be conducted at fortnightly intervals during the video consultation for the intervention group only.

#### Sleep quality

The 19-item Pittsburgh Sleep Quality Index will quantify sleep quality and duration over the previous month at baseline, 6 weeks and 12 weeks.^37^ The questionnaire assesses seven separate sleep components, including subjective sleep quality, sleep onset latency, sleep duration, sleep efficiency, sleep disturbances, use of sleep medication and daytime dysfunction. Each question results in a score from 0 to 3 points which are then summed to create a global score ranging from 0 to 21 points, whereby higher scores indicate poorer sleep quality. A global score of five points or greater indicates poor sleep quality.^37 38^ The Pittsburgh Quality Sleep Index has moderate to good test–retest reliability (ICC=0.70–0.86).^37^

#### Insomnia severity

The seven-item Insomnia Severity Index will be used to assess the nature, severity and impact of insomnia at baseline, 6 weeks and 12 weeks.^39^ The questionnaire evaluates the following insomnia dimensions: severity of sleep onset, sleep maintenance, early morning awakening problems, sleep dissatisfaction, interference of sleep difficulties with daytime functioning, noticeability of sleep problems by others and distress caused by sleep difficulties. The global score ranges from 0 to 28 points, whereby higher scores indicate higher severity of insomnia (0–7 points: absence of clinically significant insomnia; 8–14 points: subthreshold insomnia; 15–21 points: moderate insomnia; 22–28 points: severe insomnia). The Insomnia Severity Index has good test–retest reliability (ICC=0.84).^40^

#### Health-related quality of life

The six-item EQ-5D-5L will quantify the health-related quality of life at baseline, 6 weeks and 12 weeks.^41^ The questionnaire includes five dimensions: (1) mobility, (2) self-care, (3) usual activities, (4) pain/discomfort and (5) anxiety/depression. For each dimension, patients will identify either no, slight, moderate, severe or unable to/extreme problems. The questionnaire also includes a visual analogue scale spanning 0–100 points, which evaluates overall current health, with 0 considered the worst health one could imagine and 100 considered the best health one could imagine. A 10.5-point change is the minimum clinically meaningful difference in adults with LBP.^42^ The EQ-5D-5L has moderate to good test–retest reliability (ICC=0.73–0.84).^43^

#### Inflammatory blood markers

Within 7 days before measurements at baseline and between three and 7 days following the last exercise training session, participants will attend a commercial pathology clinic collection centre where a fasted morning (08:00–10:00) venous blood sample will be collected. Participants will be asked to refrain from eating or drinking anything aside from water 1 hour before the blood draw. Blood samples will be separated into serum/plasma by low-speed centrifugation (3000 rpm, 4°C for 10 min). Samples will be sent to a central pathology laboratory accredited by the National Association of Testing Authorities Royal College of Pathologists Australasia for testing of high-sensitivity C reactive protein (Melbourne Pathology) according to manufacturer instructions. The remaining samples will be stored in a −70°C freezer before being transferred to Deakin University, where samples will be stored at –80°C until analysis. Milliplex immunoassay kits (Millipore Corp, Billerica, USA) will measure markers associated with IVD health^44^: tumour necrosis factor alpha, interleukin 1 beta, interleukin 4, interleukin 6, interleukin 8, interleukin 12, interferon-gamma, as per the manufacturer’s recommendations. Prostaglandin E2 will be analysed by PGE2 Human ELISA Kit (Invitrogen, California, USA). Samples will be assessed in duplicate in a single batch after the study. A biological specimens’ statement is available in online supplemental appendix B.

#### Habitual physical activity

The seven-item International Physical Activity Questionnaire will quantify habitual physical activity at baseline, 6 weeks and 12 weeks.^45^ The questionnaire asks about the frequency/duration of vigorous and moderate intensity physical activity, walking and sitting over the past 7 days. Total weekly physical activity will be calculated by weighting each type of activity by its energy requirement in metabolic equivalent to produce a score in metabolic equivalent minutes. The International Physical Activity Questionnaire has moderate to excellent test–retest reliability (ICC=0.70–1.00).^45^

#### Past exercise history

The two-item Bone-specific Physical Activity Questionnaire will quantify physical activity levels by loading impact on bone.^46^ Given the rationale that IVD may similarly adapt to loading as bone, this questionnaire is relevant to the research questions in this study. The self-administered questionnaire quantifies physical activity at various ages from childhood to 12 months before the present. The second item quantifies physical activity in the last 12 months, including frequency each week. Participant responses are applied to a mathematical equation that generates a total bone-loading score, which considers ground reaction forces associated with each physical activity, frequency and years of participation. The questionnaire has been validated using accelerometery and measuring ground reaction forces in 19 physical activities.^46^

#### Recovery and treatment expectations

A three-item questionnaire will be used to assess general recovery expectations and expectations of treatment-specific benefit at baseline, 6 weeks and 12 weeks.^47–49^ To measure general recovery expectations by 6 and 12 weeks, responses are rated from 0 to 10 points, where 0 indicates no improvement and 10 indicates complete recovery. To measure treatment-specific expectations of the intervention, responses are rated from 0 to 10 points, where 0 indicates not at all helpful and 10 indicates extremely helpful.

#### Activity-specific beliefs

A six-item questionnaire will be used to assess the beliefs of safety towards specific physical activities at baseline, 6 weeks and 12 weeks. Questions one to four relate to the current and past involvement in a range of physical activities, while questions 5 and 6 evaluate the belief in safety. Question six asks the respondent, ‘Do you think the following exercises/movements are safe for you to do?’ Responses to 20 common physical activities are rated on a five-point Likert scale from definitely no to definitely yes. A 10–15 s video accompanies each activity to provide respondents with a clear reference. For activities perceived as unsafe (responses of definitely no and probably no), two additional questions will explore the meaning of this belief and the subsequent impact this has on participation.

#### Kinesiophobia

The 11-item Tampa Scale for Kinesiophobia will be used to quantify kinesiophobia at baseline, 6 and 12 weeks.^50^ Responses to questions relating to fear of movement, fear of physical activity and fear avoidance are rated on a four-point Likert scale from strongly disagree to strongly agree. Higher scores indicate greater levels of kinesiophobia. The Tampa Scale for Kinesiophobia demonstrates excellent test–retest reliability (ICC=0.91).^51^

#### Depression, anxiety and stress

The 21-item Depression Anxiety and Stress Scale will be used to measure depression, anxiety and stress at baseline, 6 and 12 weeks.^52 53^ Seven items on a four-point scale measure each factor of depression, anxiety and stress, where 0 indicates never and 3 indicates almost always. Scores range from 0 to 21 points on each subscale, with scores multiplied by two, and higher scores indicate greater levels of depression, anxiety and stress, with additional subscales for each construct. The Depression Anxiety and Stress Scale has good to excellent test–retest reliability (ICC=0.82–0.90).^52^

#### Pain catastrophising

The 13-item Pain Catastrophising Scale will measure pain catastrophising at baseline, 6 and 12 weeks.^54^ Responses are rated on a five-point scale, where 0 indicates not at all and 4 indicates all the time. Higher scores represent greater levels of pain catastrophising. The Pain Catastrophising Scale has good to excellent test–retest reliability (ICC=0.80–0.93).^54^

#### Pain self-efficacy

The 10-item Pain Self-efficacy Questionnaire will measure self-efficacy towards managing pain at baseline, 6 and 12 weeks.^55^ Responses are rated on a seven-point scale, where 0 indicates not confident at all and 6 indicates confident. Higher scores represent greater levels of pain self-efficacy. The Pain Self-Efficacy Questionnaire has excellent test–retest reliability (ICC=0.92).^56^

#### Social support

The 12-item Multidimensional Scale of Perceived Social Support will be used to measure perceived social support at baseline, 6 and 12 weeks.^57^ Responses are rated on a seven-point Likert scale and range from very strongly disagree to very strongly agree. Higher scores indicate greater levels of social support, with three subscales representing support from family, friends and significant others. The Multidimensional Scale of Perceived Social Support has good test–retest reliability (ICC=0.85).^58^

#### Pressure pain thresholds, exercise-induced hypoalgesia and expectations

Pressure pain thresholds will be used to examine hypersensitivity using a digital algometer (Commander Echo, J Tech Medical Industries, Salt Lake City, USA) at the forearm, paraspinals and calf using standard and reliable protocols at baseline and 12 weeks.^59 60^ Manual pressure will be applied using a 10 mm diameter rod at a rate of approximately 10 N/cm^2^ per second until the participant perceives the pressure as pain. The protocol will consist of two trials on each muscle region (left, right, left, right) before proceeding to the next region (ie, forearm, lumbar paraspinal, then calf) with 20 s rest between each trial. Test–retest reliability is excellent for pressure pain thresholds (ICC: wrist=0.81–0.97, leg=0.96–0.98, neck=0.92–0.98, back=0.94–0.99).^61^ Participants will receive neutral language instruction and complete familiarisation for one trial at each site following the procedures described above.^62^ To test endogenous analgesia (descending pain inhibition) in response to exercise, pressure pain thresholds will be reassessed following a 3 min isometric wall squat with feet at shoulder width and knee joint angle of 100 degrees using an established protocol.^59 63^ LBP intensity (visual analogue scale) will be evaluated immediately before and after the wall squat to determine a change in endogenous hypoalgesia. Participants will also record a rating of perceived exertion (6–20 points) following the exercise.^64^ Following pressure pain thresholds, participants will complete a two-item questionnaire to determine expectations of the wall squat on changes in pressure pain thresholds and LBP intensity.^62^

#### Adverse events

Participants will be instructed to inform the research team immediately should any adverse events occur. Moreover, participants allocated to exercise training will be asked about adverse events during a fortnightly video consultation (Zoom Video Communications, California, USA) with a study team member. Serious adverse events will be defined as any untoward medical occurrence that results in death, is life-threatening or requires hospitalisation. Non-serious adverse events will be defined as any other untoward medical occurrence. Adverse events will be classified as treatment-related if they are definitely or probably related to the exercise training intervention.

#### Participant feedback

At the end of the intervention, participants will complete an in person 60 min semistructured qualitative interview with a member of the research team with guiding questions centred around two key themes: (1) feasibility of the programme and (2) running-related beliefs.

#### Physical assessments

Both anthropometry and physical capacity (muscle flexibility, strength and power) will be quantified at baseline only. Outcomes will facilitate safety regarding exercise prescription and serve as potential confounding factors in statistical analyses. The following measures will be obtained using standard techniques: body mass, height, blood pressure, sit and reach,^65^ max single-leg calf raise,^66^ 30-second single-leg sit to stand,^67^ triple hop for distance^68^ (online supplemental appendix C).

### Participant timeline

**Figure 1:**
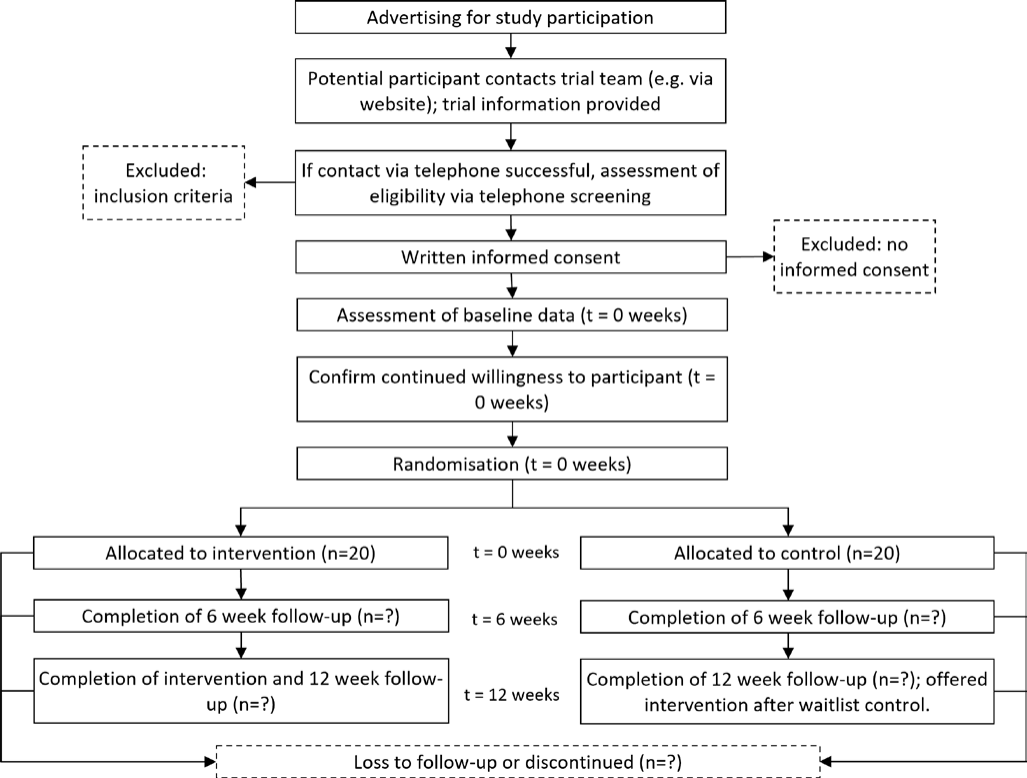
Study flow diagram.

Recruitment commenced on 3 October 2022 and will continue until 40 participants are enrolled. All participants will attend face-to-face data collection at baseline, 6 and 12 weeks post baseline (figure 1). Participants allocated to exercise training will commence the week of baseline data collection and continue until 12-week postbaseline data collection.

### Sample size

In line with recommendations,^69^ the current study was designed to detect the smallest effect size of interest between the intervention and control for each primary outcome (IVD T2, pain intensity and disability) at 12 week follow-up assuming an α of 0.05, β of 0.8 and adjustment for test–retest reliability.^70^ To detect a between-group net difference in IVD T2 of d=0.183 based on a minimum detectable change of 5.1 ms,^30^ SD of 27.9 ms^30^ and test–retest reliability of r=0.97,^30^ 28 total participants are required (n=14 per group). To detect a between-group net difference in pain intensity of d=1.00 based on a clinically meaningful change of 20 mm,^34^ SD of 20 mm^71^ and the most conservative test–retest reliability of r=0.57,^35^ 16 total participants are required (n=8 per group). To detect a between-group net difference in disability of d=0.52 based on a clinically meaningful change of 10 points,^72^ SD of 19.2 points^73^ and test–retest reliability of r=0.83,^72^ 20 total participants are required (n=10 per group). To account for a conservative attrition rate of 30%, despite retaining 80% of CLBP participants throughout our previous 6 month randomised controlled trial,^10^ 40 total participants (n=20 per group) will be recruited to detect the three primary outcomes smallest effect estimates of interest. All power calculations were conducted using G*Power (V.3.1.9.7).^74^

### Recruitment

Potential participants will be initially contacted via web-based advertisements (eg, social media) and directed to a Deakin University website with a plain language statement and an option to express interest. Those who express interest will be screened via phone, and eligible participants will provide written informed consent before enrolment.

### Allocation

Participants will be randomly assigned (1:1) using block randomisation with random block lengths (between 2 and 6 per block) and stratification for sex to either intervention or waitlist control. A team member without contact with participants will obtain and employ the randomisation schedule (creating sequentially numbered, opaque, sealed envelopes). The randomisation schedule will be developed using the ‘blockrand’ package in R V.4.1.2 (https://www.r-project.org/).^75^

### Blinding

Given the nature of the intervention, neither participants nor team members administering the intervention will be blinded to treatment allocation. All objective outcomes of efficacy (eg, IVD health, pain-related inflammatory blood markers) will be collected by researchers blinded to treatment allocation. For subjective efficacy outcomes (eg, pain intensity, disability, kinesiophobia), the participant is considered the assessor and, therefore, not blinded to treatment allocation. Before data processing of objective measures of efficacy (eg, IVD T2), each participant will be allocated a random code to researchers who are blinded to treatment allocation. During data processing, researchers will also be blinded to the order of observations (baseline, 6 and 12 weeks post baseline). Following data processing, all biostatistical analyses will be blinded to treatment allocation and only revealed once all a priori analyses are completed.

### Data management

All data will be deidentified and stored on password protected servers at the institutions involved in the current study. All study-related electronic documents will be archived indefinitely at the institutions involved at the end of the study, which is in line with current ethical requirements. Only the researchers involved in this project will have access to the data collected.

Participation in the study is entirely voluntary, and withdrawal of consent will not jeopardise the relationship between the participant and any institution involved with the current project; thus, participant data are expected to be genuine. To minimise missing data, follow-up in-person testing will be booked at least 3 weeks in advance, and regular reminders will be provided to participants. Automatic electronic reminders will occur should the participant not complete follow-up questionnaires within 24 hours of receipt of the original notification. If questionnaires remain incomplete 48 hours after receipt, the participant will receive a phone call from a team member. Where data are deemed missing, linear mixed models can account for the absence of these data, and appropriate sensitivity analyses will be conducted to ensure all analyses meet these assumptions.

### Statistical methods

Linear mixed models with random effects (participants) and an intention-to-treat approach will be used to evaluate within-group and between-group changes by time. All linear mixed models will allow for heterogeneity of variance according to study date and employ restricted maximum likelihood estimations. An α of 0.05 will be adopted for all analyses.

### Monitoring

No data monitoring committee will be employed, given the low-risk nature of the intervention examined. Subsequently, no independent auditing will occur throughout the trial.

## Ethics and dissemination

### Research ethics approval

Ethics approval was provided by the Deakin University Human Research Ethics Committee (ID: 2022-162) on 26 September 2022.

### Protocol amendments

Any protocol amendments will be communicated via updates to the Australian New Zealand Clinical Trials Registry (ACTRN12622001276741). A section on protocol amendments will also be included in any publications from this work.

### Consent

All potential participants will be screened initially via phone with a research team member to determine their eligibility. All eligible participants will be required to provide informed consent on reviewing the participant information and consent form (online supplemental appendix D). Participants will retain a copy of the forms, including information regarding their ability to withdraw their consent if desired. This consent process will be uniform across all participants. The right to withdraw consent without question or consequence is outlined in the patient information and consent form, which will be provided to patients electronically before and once again after enrolment.

### Confidentiality

Each participant will be allocated a study-specific code. However, all data will remain reidentifiable for cross-checking data entry and disseminating individual data to participants after the study. The codes will be stored electronically on password protected servers at the involved institutions, and only research staff involved in this project will have access. The codes will be kept separate from personal data collected on the participants.

### Ancillary and post-trial care

On completion of the 12-week study, participants in the waitlist control will be offered the study intervention under the supervision of the study team. No other follow-up is currently planned beyond the 12-week study period.

### Dissemination policy

Authorship of outputs from this research will be in line with the International Committee of Medical Journal Editors guidelines. Findings from the primary outcomes of this study will be reported in journal articles and student theses, which will include results regardless of the direction or magnitude of the effect. The results will also be presented at leading national and international conferences, clinical forums and to other relevant health professionals and stakeholders and the participants. The dissemination of research results will only consist of aggregated data; individual participants will not be identified. All participants will be able to elect to receive a brief report of their study results via email after the study. If the participant expresses interest in the study results, they can request to have a copy of published articles sent to them.

## Discussion

This will be the first study to evaluate whether running exercise can improve IVD health. Surgical options are commonly implemented to treat poor IVD health yet are associated with additional risk. Therefore, identifying conservative options to improve IVD health has the potential to reduce the marked burden of LBP.

## Data Availability

Data sharing not applicable as no datasets generated and/or analysed for this study. Protocol only.
