# Supplementary material for: Assessing safety and treatment efficacy of running on intervertebral discs (ASTEROID) in adults with chronic low back pain: protocol for a randomised controlled trial
Source: BMJ Open Sport Exerc Med. 2023 Jan 17;9(1):e001524. doi: 10.1136/bmjsem-2022-001524 (PMC9853241; doi:10.1136/bmjsem-2022-001524)
Supplement: Supplementary data [file bmjsem-2022-001524supp001.pdf]

APPENDIX A: Educational materials

Email 1: Ideal running speed

Hello [first_name],

We hope you're going well with the program so far. Don't hesitate to get in touch if you do need anything or have any issues as we go along.

On top of the Zoom check ins, we want to share with you some educational content throughout the program. As the saying goes , "knowledge is power" and this definitely applies when it comes to managing low back pain.

We've picked a few key topics to cover that will help you get the most out of your running program.
It should only take a few minutes to read through each email, and we hope this will add value to your training experience.

This week's email is around choosing the ideal running speed, or more accurately, whether there is an ideal running speed.

Throughout the 12 weeks we recommend you run at a comfortable pace with a maximum speed of 6 mins/km, which is equal to 10 km/h. We have made this recommendation based on prior research that has looked at which running speeds are most beneficial for our spinal discs (1).

This doesn't mean that jogging quicker than 6 min/km is bad for you, but for this study it will help us test whether we see an improvement at this speed.

Don't worry too much if you go a little bit quicker at times, but do your best to keep it under 10km/h, which for most people is a slow to moderate pace.

And if you do run slower than 6 min/km, that's completely OK. Actually, it's probably a good thing.

Like all exercise, when starting a new running program, we advise people to start slow and build up gradually. This is the best way to minimise the risk of injury or a flare up of pain.

On your next run, see if you can check your running speed on the Runkeeper app and see how you're going. Aim to run at a comfortable pace and keep it under 6 min/km for most intervals.

We'll be keen to hear how you go at our next meeting!

Talk soon,

The ASTEROID Team

References:
1. Belavý DL, Quittner MJ, Ridgers N, Ling Y, Connell D, Rantalainen T. Running exercise strengthens the intervertebral disc. Sci Rep. 2017 May;7(1):45975.

Email 2: Footwear selection

Hello [first_name],

We just wanted to touch base and send through some advice on footwear selection.

It can all get a bit confusing with all the shoe brands out there. Not to mention all the different types of shoes to match different surfaces and running styles.

What you might find interesting is that what shoes you wear has very little bearing over your likelihood of injury.

There are countless research papers that have looked at running shoes and injury risk and most find that the type of running shoe you wear doesn't change your risk of injury (1).

It's kind of nice to know that you don't need to go out and buy expensive running shoes to do this program. In fact, this study (2) found that the more people pay for running shoes, the more injuries they'll have. Perhaps the reason for this is doing too much too soon, which is a common reason for getting an injury.

. You might have noticed that this program builds up slowly from week to week. This is to reduce your risk of injury so you don't need to worry about finding the 'right' shoes.

You might be wondering if there's no ONE best shoe type, then what should you look for in a running shoe?

Here are a few recommendations:

1) Firstly, if you're happy with the pair of shoes you currently have then stick with them. Sometimes this is the safest bet as you know what they're like and that they suit your foot.
2) Secondly, if you are ready for a new pair (I mean who doesn't like new shoes?), the most important factor is finding something comfortable. That means trying it on in store to see how it feels on your foot. Does is feel good under the arch and around the heel, how about across your toes and the front of your foot. Don't be afraid to go for a walk and do some hopping on the spot. Some people like to go up half a size for their running shoes to allow for a bit of room if your feet swell a little with heat.
3) Thirdly, if you can go for a run in those shoes that's even better. While it's not essential, you might find that running in new shoes feels a bit different to just walking around the store. Therefore, have a look for a store with a treadmill and see if you can go for a quick jog. If it still feels comfortable, you're probably on to a winner.

That's it! Three simple tips to help guide you find the right shoe.

At the end of the day, if the shoe fits then go with it. If the shoe looks good on you when you're running the streets, even better!

Hope this helps, but if you do have any questions just reply to this email and we'll be in touch.

Happy running!

The ASTEROID Team

References:
1. Malisoux L, Theisen D. Can the "Appropriate" Footwear Prevent Injury in Leisure-Time Running? Evidence Versus Beliefs. J Athl Train. 2020 Dec 1;55(12):1215–23.
2. Marti B, Vader JP, Minder CE, Abelin T. On the epidemiology of running injuries. The 1984 Bern Grand-Prix study. Am J Sports Med. 1988 Jun;16(3):285–94.

Email 3: Is it safe to run following a back Injury?

Hey [first_name],

One of the questions we often get from people following a back injury, is ‘is it safe for me to run’?

Given the amount of misinformation out there, we wanted to help clear this up.

First and foremost, we’ve gone through a thorough screening process to ensure you are safe to participate in this program. By running through your medical history, conducting physical tests and completing MRI scans, we can be confident that this program is suitable for you.

But in general, how safe is running for people with back pain?

The answer might surprise you.

It appears that people who run regularly report less back pain than those who don’t. This study (1) found that runners tend to report lower levels of back pain than the general population. And the levels of back injuries in runners ends to be much lower than knee or ankle injuries. The authors concluded that running may provide a protective effect for our spines.

This could be due to lots of possible reasons.

The authors of this study (2) believe that running may be beneficial for our spinal discs. This could be the reason that runners have less pain. With your help, this is something we are trying to confirm in our study… does running make your spinal disc healthier?

Another study (3) compared MRI scans of 79 runners and found that people who run regularly have healthier spines than people who don’t run at all.

We also know that aerobic exercise like running can reduce inflammation, help with weight loss and strengthen your leg muscles. For all these reasons (and more!) we think running is safe and beneficial following a back injury.

With our help we hope that you’ll find running beneficial too. The key is to build up gradually and listen to your body.

This doesn’t mean that you won’t have any back pain at all or you won’t get niggles and flare ups doing this program. If you do get a sore back from running let us know and we can help manage it to get your back on track (more on that next week!).

The key takeaway: running is safe for more people with back pain when you build up gradually.

If you find this information helpful, you might want to share it with your family and friends or even your doctor? We want to spread the word that running is OK for people following a back injury and may play a part in getting better.

The ASTEROID Team

References:

1. Maselli F, Storari L, Barbari V, Colombi A, Turolla A, Gianola S, et al. Prevalence and incidence of low back pain among runners: a systematic review. BMC Musculoskelet Disord. 2020 Dec;21(1):343.
2. Belavý DL, Albracht K, Bruggemann GP, Vergroesen PPA, van Dieën JH. Can Exercise Positively Influence the Intervertebral Disc? Sports Med. 2016 Apr;46(4):473–85.
3. Belavý DL, Quittner MJ, Ridgers N, Ling Y, Connell D, Rantalainen T. Running exercise strengthens the intervertebral disc. Sci Rep. 2017 May;7(1):45975.

Email 4: Dealing with setbacks

Hey [first_name],

Last week we talked about how running was safe for people with back pain.

Its important to also consider that the occasional setback is normal when you are active.

Little niggles, a flare up in pain, a sore ankle or dodgy knee, these things are all part of life and being active.

However, with the right plan in place they don't need to be more than a temporary setback.

Firstly, to help minimise injuries and soreness in the first place, we recommend having a rest day between runs. While this isn't always possible, if you can fit in a rest day it will give your body time to recover after each run.

We also recommend building up gradually. This is why we suggest only increasing one stage of the program per week even if it feels a bit easy at first. We've also aimed to make the increments from one stage to the next really achievable by keeping it to 15 seconds each week.

With that said, it is normal to have some soreness during or after running sessions. Ideally, any soreness will clear up within 24-48 hours so you can get on with the next session.

However, if you do have an increase in pain or an issue that hangs around for more than a day or two, please let us know.

Depending on how you're going, we might recommend you have a break from running or reduce your training volume. This may mean temporarily going back to a previous stage of the training program.

The important thing to remember is that setbacks are only temporary and progress doesn't always happen in a straight line. Sometimes you need to take one step back to take two steps forwards.

So, try not to lose perspective if you do suffer a temporary setback. And remember we're in your corner if you do need any support or guidance.

If you do have any other questions for us, please let us know, either via email or on our next Zoom call.

Talk soon,

The ASTEROID Team

APPENDIX B: Biological specimens

The trial will not involve genetic or molecular analysis of biological specimens derived from humans. However remaining sera will stay de-identified and stored for future use at Deakin University. If remaining specimens collected by this project are to be used for another analysis or study, the researchers would seek ethical approval for this.

APPENDIX C: Table 1. Baseline physical assessment details.

**Body mass**: Participants will be asked to remove their shoes and any excess clothing (e.g. coat, scarf, heavy jackets; light jumpers are permitted). Body mass will be taken using standard floor scales. Participants will stand still on the scales and the assessor will record the final reading once it stabilises. Scores will be recorded in kg.
**Height**: Height will be measured using a wall mounted measuring tape. The participant will be asked to stand against a wall and encouraged to take a deep breath in and out. The assessor will record the height from the top point of the head at the end of the exhale. Scores will be recorded in cm.
**Blood pressure**: The participant will be seated with their left arm supported on a surface. The assessor will place the automatic blood pressure cuff slightly above the cubital fossa, with the artery index marker in line with the brachial artery. If the reading is greater than 140/90 mmHg, the participant will be asked to rest for two minutes before repeating the test. If the second reading is above 140/90 mmHg, the participant will be asked to seek approval from their general practitioner prior to enrolment. Scores will be recorded in mmHg.
**Sit and reach:**[63] The assessor will provide a demonstration. Participants will be seated on the floor next to a wall with both legs outstretched. They will be asked to lean forward as far as they can while keeping their knees straight. The assessor will measure the position of the participants finger tips in relation to the top of the toes by placing the ruler along the wall for steadiness. The soles of the feet should be perpendicular to the ground. The participant will need to hold their hands steady (i.e. no bouncing) for 2-seconds for a measurement to count. Participants will have three attempts, with only the highest score being recorded. Scores before the toes will be negative, reaching exactly the toes will be zero and beyond the toes will be positive. Scores will be recorded in cm.
**Triple-hop for distance**:[66] The assessor will provide a demonstration. The participant will be asked to stand on one leg with the toes behind a line marked on the floor. The participant will hop three times in a row on the same leg, as far forward as they can. Total distance for the three hops will be measured as the distance between the front of the participants foot in the start and finish positions. The assessor will record the score for that side before completing the test on the other side. Scores will be recorded in cm.
**Max single leg calf raise**:[64] The assessor will provide a demonstration, highlighting the importance of raising up not forwards. The participant will be asked to stand on one foot, as close as they can to a wall. Hands can be on the wall for stability. The participant will be instructed to raise up onto their toes as high as they can and then back down to the floor, while maintaining a straight leg. The pace will be maintained at two seconds up and two seconds down, which the assessor will count out loud. Once the participant loses more than 2cm height at the top of the calf raise, or are unable to maintain the speed with cueing (e.g. receives one warning and after this decreasing speed will likely be due to fatigue) the test will be stopped. The assessor will record the maximum number of full range single leg calf raises for that side before completing the test on the other side.
**30-sec single-leg sit-to-stand**:[65] The assessor will provide a demonstration, highlighting the importance of controlling the decent. The participant will be asked to sit on a solid chair with the chair back against a wall. The participant will be instructed to stand using one leg only with their arms across their chest. If the participant loses balance they may place the other foot on the group to maintain balance only, before continuing the test on the same leg. The assessor will time 30 seconds, providing a 5-second warning at the 25 second mark. If the participant is halfway through a repetition at 30-seconds, it will not count. The assessor will record the number of sit-to-stands on that side before completing the test on the other side.

APPENDIX D: Plain language statement and consent form

PLAIN LANGUAGE STATEMENT AND CONSENT FORM

TO: Participant

Plain Language Statement

Date: 22/11/2022
Full Project Title: Can exercise improve intervertebral discs in individuals with back pain?
Principal Researcher: Dr Patrick J Owen **(Deakin University)**
Student Researcher: Claire L Samanna **(Deakin University)**, Christopher Neason **(Deakin University)**, Emma A Craige **(CQUniversity)**
Associate Researcher(s): Prof Daniel L Belavy **(Hochschule für Gesundheit, Germany)**, Prof Ulrike H Mitchell **(Brigham Young University, United States of America)**, A/Prof Steve Bowe **(Victoria University of Wellington, New Zealand)**, A/Prof David Scott **(Deakin University)**, A/Prof David Connell **(Imaging@Olympic Park)**, Dr Niamh L Mundell **(Deakin University),** Dr Clint T Miller **(Deakin University)**, Dr Jamie L Tait **(Deakin University)**, Dr Luana C Main **(Deakin University)**, Dr Grace E Vincent **(CQUniversity)**, Dr Matthew Clarkson **(VUUniversity)**, Scott D Tagliaferri **(Deakin University),** Romina Gollan **(University of Cologne).**

This Plain Language Statement and Consent form is 11 pages long. Please make sure you have all the pages.

1 Your Consent

You are invited to take part in this research project.

This Plain Language Statement contains detailed information about the research project. Its purpose is to explain to you as openly and clearly as possible all the procedures involved in this project so that you can make a fully informed decision whether you are going to participate.

Please read this Plain Language Statement carefully. Feel free to ask questions about any information in the document. You may also wish to discuss the project with a relative or friend or your local health worker. Please feel free to do this.

Once you understand what the project is about and if you agree to take part in it, you will be asked to sign the Consent Form. By signing the Consent Form, you indicate that you understand the information and that you give your consent to participate in the research project.

2 Purpose

The overall aim of this study is to understand whether exercise can improve intervertebral disc health in individuals with low back pain.

If you are eligible for this study, you will be assigned, at random, to one of two groups. Both groups will receive low back magnetic resonance imaging (MRI) scans at Imaging@Olympic Park (Melbourne) on three occasions (baseline, six weeks following baseline and 12 weeks following baseline) over the course of the study. All participants will complete a series of questionnaires at baseline, 6- and 12-week follow-up. All participants will also complete an assessment of pressure sensitivity threshold at baseline and 12-week follow-up. Additionally, both groups will provide a blood sample at a community-based pathology collection centre (Melbourne Pathology) at baseline and 12-week follow-up.

One group will be provided with an assessment and individual aerobic exercise program you can complete from home under the guidance of an Exercise and Sports Science Australia accredited exercise physiologist (a tertiary trained allied health professional who specialises in the provision of exercise training for the management of chronic health conditions such as low back pain). The program includes three 30-minute sessions each week for 12 weeks consisting of interval-based walking and jogging. The intensity and progression of walking and jogging will be individualised based on your current ability.

The second group will be asked to manage their low back pain as usual (e.g. general practitioner management, over-the-counter medications) for 12 weeks. Following these 12 weeks, you will be eligible to receive the same assessment and exercise program as group one.

To understand what changes occur in your body due to the different treatment approaches, a number of tests will be performed at the start of the study, at 6-weeks and at 12-weeks. These include MRI scans, tests of muscle strength, performance and endurance and a series of questionnaires. Also, at the end of the program, you will be asked to complete an online 60-minute interview via Zoom to gather your feedback on how you managed completing the exercise program.

3 Methods and Demands: what you will be asked to do

If you participate in the study, you will undergo **MRI scans at Imaging@Olympic Park,** Melbourne, Australia on three occasions. These scans will be performed at least four hours since waking in the morning. If you wish to participate in the study, it is important that you can **commute to Imaging@Olympic Park** and that you can **commit to the exercise training program** outlined here.

Both groups

- Three in person MRI scans at Imaging@Olympic Park, Melbourne at baseline, 6- and 12-week follow-up
- The MRI testing session will take approximately 60 minutes, allowing for 20 minutes of sitting time prior to the scan (20-30 minutes)
- Two pressure sensitivity threshold tests after your MRI at baseline and 12-week follow-up.
- Two in-person blood samples provided at a community-based commercial pathology collection centre of your choice at baseline and 12-week follow-up. This process should take 15 minutes on each occasion.
- Online questionnaire at baseline, 6- and 12-week follow-up. The questionnaire will be completed during your sitting time at each MRI scan visit.

Group 1 (intervention group)

- You will receive a 30-minute initial physically assessment at Imaging@Olympic Park immediately after your first low back MRI scan by an accredited exercise physiologist.
- The accredited exercise physiologist will ask you some health history questions, complete a low back assessment, determine your muscle strength and evaluate your aerobic capacity. Please ensure you wear exercise appropriate clothing for this assessment including runners.
- The accredited exercise physiologist will arrange weekly follow-up phone calls to monitor your progress throughout the exercise program. These will take approximately five minutes per call.
- You will receive an exercise program which is designed to be completed within the community, using phone app to track your exercise sessions.
- The program will be a progressive aerobic exercise program that consists of walking and jogging completed on three days per week for up to 30 minutes.
- The program will be individualised to match your current ability, determined at your Exercise Physiology assessment and will include walking with intervals of jogging.
- Your exercise sessions will be tracked via a free phone app called ‘Run Keeper’. You will be guided how to download and use this app during your testing session.
- During your weekly phone calls, you and your Exercise Physiologist will together determine when your program is ready to be progressed to the next level.
- You will be asked to record your pain levels and effort level after the completion of each exercise training session and at 24 hours following the session.

Group 2 (control group)

- You will be asked to manage your low back pain as usual.
- During the 12-week follow-up, you will be asked to not start any new exercise program.
- After your third MRI scan at 12-week follow-up, you will be eligible for the same exercise training program provided to Group 1 (see above).

You cannot choose which group you will participate in and will be allocated at random by an external member of the research team.

Even if your pain resolves, it is expected that you continue with the treatment program.

If you are eligible for the study, you will be randomised (assigned by chance) to one of the two groups. If you are not **certain that you can attend the MRI scans, provide blood samples** or **complete the exercise training** as listed above for the **full 12-week period** of the study, then you **should not participate in this study**.

The following tests will be performed at Imaging@Olympic Park (AAMI Park, 60 Olympic Boulevard, Melbourne, VIC 3004, Google Maps: https://goo.gl/maps/xMGSN):

- A **magnetic resonance imaging** (MRI) scan will be used to measure the discs, muscles, bones, fat content, water content and your ability to contract the muscles of your back and stomach.
- Measurements of your **body size** such as height and weight.
- Your **pressure sensitivity threshold** will be determined using a pressure rod place on your forearm, lower back and calf before and after a 3-minute wall squat exercise. You will be asked to complete a small questionnaire on your expectations on change in pain and overall effort level for the exercise.

For those is group 1 (intervention group), you will also complete the following tests.

- Your **physical function and capacity** will be measured with a range of physical tests. An individualised low back assessment will be completed prior to the following physical tests. The sit and reach test involves sitting on the floor/bed and reaching forward as far as you can to measure your low back and hamstring flexibility. The next two tests will measure you lower body strength: 1) the single leg heel raise test will involve standing on one leg (using the wall to balance) and raising up on your toes as many times as you can and repeating on the other leg; 2) the 30-second single leg sit to stand test will involve standing up from a chair using one leg only as many times as you can in 30 seconds, before repeating on the other leg. The three-hop test will test your lower body power and will include hopping forwards on one leg, three times in a row and the distance covered for all three hops will be measured.
- Your **baseline endurance capacity** will be measured outdoors at the Imaging@Olympic Park site. You will undertake a walk warm up for two minutes followed by jogging for up to two minutes. You will guide how long you jog for (up to two minutes) with the option to stop at any point during this test. This test will guide the starting point for your exercise program.

Directions to the scanning facility:

- By car: When coming down Swan St (from Richmond end) and after crossing Punt Road, turn left at first set of lights in Swan St (Entrance F). There is parking on the grounds which is free of charge for people attending the scanning centre. Bring the parking ticket in for validation when you come.
- By tram: Route 70, get off at Stop 7D AAMI Park (~150m from scanning centre)
- By train: Richmond Train Station (~600m from scanning centre)

Some things to keep in mind on the day of scanning:

- The actual scan will take approximately 30-40 minutes followed by a 30 minute Exercise Physiologist assessment, but you should allow time for possible appointment delays
- Please ensure you have comfortable clothing that does not restrict your movement and that there are no metallic objects on your clothes or underwear, otherwise you will be given a gown to wear for the scanning. Please note that you cannot take any metallic or electronic items into the scanner. Please refrain from wearing jewellery where possible especially earrings, most other piercings will not affect the images.

Please note: as part of standard protocols at Imaging@Olympic Park, your MRI scans will be reviewed by a radiologist. If there are any findings that require medical review, you will be referred back to your GP for review prior to continuing with the study.

4 Potential benefits to participants

We cannot guarantee or promise that you will receive any benefits from this project.

However, we expect that participants in both groups will benefit from their involvement in the treatment program. We also expect improvements in pain, function, muscle function, muscle strength.

Furthermore, at the end of the 12 week project, if you wish we can provide you with the results from your MRI and physical function testing. This is access to information on your own body that you would not otherwise have.

It is possible that in the course of the study we might obtain information, such as from MRI, which might be of clinical significance. In this case we will inform you and advise you to talk to your GP.

5 Potential risks to participants

There are, however, some potential risks of the treatment program and/or testing program which you should consider before deciding to commit to this study. Some groups of people are at higher risk of negative side effects due to the study (e.g. pregnancy, certain types of metallic objects in some objects in some body regions, history of seizures, history of serious head injury) and will be excluded from the study.

- Intervention group: It is possible that some participants will experience some minor discomfort due to their performance of the exercise intervention. General muscle soreness due to exercise is possible and common. This is a normal response to progressive overload exercise and dissipates within a few days. There is a small possibility that some participants in either group will experience deterioration in their condition over time that is not necessarily related to the intervention.
- Magnetic resonance imaging (MRI): MRI scanning has been in use as a medical imaging tool for many years and with proper safety controls is commonly regarded by clinicians as a safe procedure. It does not employ ionising radiation (such as x-rays). It does however entail exposure to electromagnetic fields (EMF) which are much higher than

levels recommended by international safety guidelines for general exposure (though still within limits of special guidelines for MRI scanning). Very occasionally, these EMFs may cause some tingling or heating sensations. These effects do not persist after scanning and have no known long term impact on health. Your MRI exposure will be carefully controlled to avoid such effects, and you will be constantly monitored for any signs of these effects and may direct us to stop the scan at any time if you experience uncomfortable sensations. The staff on duty will answer any queries you might have on the day, or if in doubt, call our department before your appointment. Some people cannot undertake MRI scanning. People for whom MRI scanning may be potentially unsafe will be excluded from the study. You will be asked to complete a safety questionnaire by the radiographer on duty and they will determine whether you can safely enter MRI.

- Blood draw: This may be perceived as unpleasant by some participants and may cause minor pain or bruising at the site of the needle entry. To reduce risk, this will be completed by a qualified phlebotomist at an accredited commercial collection facility following established clinical guidelines.
- Physical function and performance: you may experience some muscle soreness in the days after testing. This is a normal response of the body to strength and endurance testing.
- Endurance capacity: muscle soreness due to the exercise is possible. This is a normal response of the body to exercise and dissipates after a few days.
- Body size and mass measurements: no known risks
- Pressure sensitivity threshold test will cause some temporary discomfort; however, the test is over quickly and is not expected to cause any pain once completed.
- Questionnaires: no known risks

There may be additional unforeseen or unknown risks.

6 Expected benefits to the wider community

The findings of this study will help the wider community to treat lower back pain. Back pain is a major public health concern that presents the greatest costs to Australian society, above that of cardiovascular disease, diabetes, cancer and other diseases, in terms of disability and lost productivity. The current study will deliver information that will improve the management of spinal pain conditions.

7 How privacy and confidentiality will be protected

Any information obtained in connection with this project and that can identify you will remain confidential. It will only be disclosed with your permission, subject to legal requirements.

Forms with identifying information will be stored separately from other study information. For the purpose of treatment and testing appointments, we will use your name in our internal booking systems. Any identifying information stored in electronic form will be stored on a password protected computer.

A unique code will be used on all forms and data collected from you, and not with your name or any other identifying information. These (de-identified) data will be stored on a password protected server.

Only the investigators will have access to the data. Sharing of data with investigators outside of Deakin University will occur only in a coded, anonymised way and no identifying or personal information will be shared.

Your general practitioner will be informed when necessary of any health-relevant events that occur or findings (such as from MRI) that are obtained due to your participation in the study.

Otherwise, none of the information provided will be made public in any form that would reveal your identity to an outside party, thus all participants will remain anonymous.

Questionnaires that are filled out online will be completed using only your unique study code. This will ensure we can collate your data internally at Deakin University, but your personal identifying information will not be used for these online questionnaires.

Information collected for, used in, or generated by this project may be used for another purpose by the researcher for which ethical approval will be sought. At this time, there are no plans to use the data beyond the parameters discussed herein.

Information on paper copy, computer or CD will be stored for 15 years from the date of publication. After a period of 15 years from the date of any publication of the results from the study, paper copies of your individual responses will be disposed of in the interests of limiting physical space taken up by the records. Electronic copies of all de-identified data will be retained indefinitely and in accordance with governing ethical research practice will be made available in an open access data repository to promote access to the benefits of research. In accordance with the Freedom of Information Act 1982 (VIC), you have the right to access and to request correction of information held about you by Deakin University.

8 Dissemination of the research results

If you wish, a brief report via email or the post of your results can be sent to you at the completion of the study. If you express interest in the study results, we can send you copies of published articles via email or post. You can indicate this on the consent form.

The research team will disseminate the findings from this project to relevant agencies and health care professionals that could potentially benefit from these findings. The study results will also be presented at national and international conferences, and will appear in our annual reports and newsletters. The work from this project will also be submitted for publication to international peer-reviewed scientific journals. Should research students become involved in the project, we expect results of the study to appear in their theses. This process will allow the results to be accessed by a large number of health professionals worldwide. Once published, we will also send relevant papers to the key stakeholders in this field.

9 How the research will be monitored and conducted?

There are a series of researchers involved in this project. The primary co-ordination and implementation of the project including participant recruitment and screening will occur at Deakin University. Here we describe the role of each person in the project:

- Dr Patrick J Owen: project lead; student supervision (musculoskeletal health and exercise training expert)
- Prof Daniel L Belavy: study design and interpretation of findings (intervertebral disc and exercise training expert)
- Prof Ulrike H Mitchell: study design and interpretation of findings (intervertebral disc and exercise training expert)
- A/Prof Steve J Bowe: biostatistics (biostatistics expert)
- A/Prof David Scott: study design and interpretation of findings (musculoskeletal health and exercise training expert)
- A/Prof David Connell: provision of MRI scans (radiology expert)
- Dr Niamh L Mundell: accredited exercise physiologist; study design and interpretation of findings (clinical exercise physiology expert)
- Dr Clint T Miller: accredited exercise physiologist; study design and interpretation of findings (pressure sensitivity threshold expert)
- Dr Jamie L Tait: study design and interpretation of findings (inflammatory blood markers expert)
- Dr Luana C Main: study design and interpretation of findings (inflammatory blood markers expert)
- Dr Grace E Vincent: study design and interpretation of findings (sleep expert)
- Dr Matthew Clarkson: accredited exercise physiologist; study design and interpretation of findings (musculoskeletal health and exercise training expert)
- Scott D Tagliaferri: research assistant; accredited exercise physiologist
- Romina Gollan: musculoskeletal physiotherapist; study design and interpretation of findings (inflammatory blood markers expert)
- Claire L Samanna: PhD candidate; accredited exercise physiologist
- Christopher Neason: PhD candidate; accredited exercise physiologist

Emma A Craige: PhD candidate
There may be additional staff and students at the Imaging@Olympic Park facility who may assist with your assessments.

10 Any payments to participants?

We do not provide payment for your participation in this project. Free parking is available at Imaging@Olympic Park.

11 Sources of funding for the research

This research is funded by Deakin University.

12 Financial or other relevant declarations

The researchers involved in the study do not have any interests, financial or otherwise, that conflict in the conduct of this study.

13 Participation is voluntary

Participation in this research project is voluntary. If you do not wish to take part, you are not obliged to. If you decide to take part and later change your mind, you are free to withdraw from the project at any stage.

Your decision whether to take part or not to take part, or to take part and then withdraw, will not affect your relationship with Deakin University. You will also have the option to withdraw your data from the research project if you wish to do so prior to publication of these data in aggregate.

Before you make your decision, a member of the research team will be available to answer any questions you have about the research project. You can ask for any information you want. Sign the Consent Form only after you have had a chance to ask your questions and have received satisfactory answers.

If you decide to withdraw from this project, please notify a member of the research team or complete and return the Revocation of Consent Form attached. This notice will allow the research team to inform you if there are any health risks or special requirements linked to withdrawing.

14 Contact details

If you require further information or wish to withdraw your participation from this project, you can contact the principal researcher:

Dr Patrick J Owen
Institute for Physical Activity and Nutrition
School of Exercise and Nutrition Sciences
Deakin University
221 Burwood Highway
Burwood VIC 3125
Australia
p.owen@deakin.edu.au

15 Complaints

If you have any complaints about any aspect of the project, the way it is being conducted or any questions about your rights as a research participant, then you may contact:

The Manager, Ethics and Biosafety, Deakin University, 221 Burwood Highway, Burwood Victoria 3125, Telephone: 9251 7129, research-ethics@deakin.edu.au

Please quote project number 2022-162

PLAIN LANGUAGE STATEMENT AND CONSENT FORM

TO: ***Participant***

Consent Form

Date: 22/11/2022
Full Project Title: Can exercise improve intervertebral discs in individuals with back pain?
Reference Number: 2022-162

I have read, or have had read to me, and I understand the attached Plain Language Statement.
I freely agree to participate in this project according to the conditions in the Plain Language Statement and can commit to the 3 month course of this project including 3 sessions of MRI testing, 2 pressure sensitivity threshold tests, 2 blood samples, an initial physical function and capacity test and the 3 month exercise program as outlined above.
I have been given a copy of the Plain Language Statement and Consent Form to keep.
The researcher has agreed not to reveal my identity and personal details, including where information about this project is published, or presented in any public form.
Please indicate below whether you agree to have your contact details stored to be invited to participate in future research.

☐ Yes, I agree to be invited to participate in future research.

Please tick the box below if you would like to receive a copy of the study results, via email, at the end of the study.

☐ Yes ☐ No

Participant's Name (printed) ……………………………………………………………………

Signature ……………………………………………………… Date ………………………..

Please mail or email this form to:

Dr Patrick J Owen
Institute for Physical Activity and Nutrition
School of Exercise and Nutrition Sciences
Deakin University
221 Burwood Highway
Burwood VIC 3125
Australia
p.owen@deakin.edu.au

PLAIN LANGUAGE STATEMENT AND CONSENT FORM

TO: Participant

Withdrawal of Consent Form

(To be used for participants who wish to withdraw from the project)

Date: 22/11/2022
Full Project Title: Can exercise improve intervertebral discs in individuals with back pain?
Reference Number: 2022-162

I hereby wish to WITHDRAW my consent to participate in the above research project and understand that such withdrawal WILL NOT jeopardise my relationship with Deakin University.

Participant's Name (printed) …………………………………………………….

Signature ………………………………………………………………. Date ……………………

Please mail of fax this form to:

Dr Patrick J Owen
Institute for Physical Activity and Nutrition
School of Exercise and Nutrition Sciences
Deakin University
221 Burwood Highway
Burwood VIC 3125
Australia
p.owen@deakin.edu.au
